# Efficient *IDUA* Gene Mutation Detection with Combined Use of dHPLC and Dried Blood Samples

**DOI:** 10.1155/2013/451298

**Published:** 2013-04-21

**Authors:** Diogo Ribeiro, Ana Cardoso, Ana Joana Duarte, Luis Vieira, Olga Amaral

**Affiliations:** ^1^Departamento de Genética Humana, Unidade I&D-P DLS, CGMJM, Instituto Nacional de Saúde Dr. Ricardo Jorge (INSA, IP), Pr. Pedro Nunes 88, 4099-028 Porto, Portugal; ^2^Departamento de Genética Humana, Unidade de Tecnologia e Inovação (UTI), Instituto Nacional de Saúde Dr. Ricardo Jorge (INSA, IP), Avenida Padre Cruz, 1649-016 Lisboa, Portugal

## Abstract

*Objectives*. Development of a simple mutation directed method in order to allow lowering the cost of mutation testing using an easily obtainable biological material. Assessment of the feasibility of such method was tested using a GC-rich amplicon. *Design and Methods*. A method of denaturing high-performance liquid chromatography (dHPLC) was improved and implemented as a technique for the detection of variants in exon 9 of the *IDUA* gene. The optimized method was tested in 500 genomic DNA samples obtained from dried blood spots (DBS). *Results*. With this dHPLC approach it was possible to detect different variants, including the common p.Trp402Ter mutation in the *IDUA* gene. The high GC content did not interfere with the resolution and reliability of this technique, and discrimination of G-C transversions was also achieved. *Conclusion*. This PCR-based dHPLC method is proved to be a rapid, a sensitive, and an excellent option for screening numerous samples obtained from DBS. Furthermore, it resulted in the consistent detection of clearly distinguishable profiles of the common p.Trp402Ter *IDUA* mutation with an advantageous balance of cost and technical requirements.

## 1. Introduction

Mucopolysaccharidosis type I (MPS I) is a rare autosomal recessive disorder resulting from deficiency of the enzyme *α*-L-iduronidase (*IDUA*, E.C. 3.2.1.76) and leads to the intralysosomal accumulation of undegraded glycosaminoglycans [1]. The gene encoding *α*-L-iduronidase (*IDUA*, OMIM no. 252800) is located on chromosome 4p16.3 and contains 14 exons [2–4]. The most common *IDUA *mutation identified in MPS I patients is p.Trp402Ter (NG_008103.1: g.20751G > A, NM_000203.3: c.1205G > A, and NP_000194.2: p.Trp402Ter) which represents at least 45% of the causal alleles in Northwestern Europe, North America, Australia, Portugal, and Spain, while in Russia, Italy, and Brazil its frequency has been estimated to be 4%, 11%, and 37%, respectively [5, 6].

Since p.Trp402Ter is the single most common causal mutation in MPS IH (OMIM no. 607014) patients of Western European origin and accounts for 60% of the alleles in unrelated Portuguese patients [7], we wanted to develop a rapid and reliable method for the convenient detection of this *IDUA* mutation (OMIM no. 252800.0001).

Denaturing high-performance liquid chromatography (dHPLC) was successfully applied to the screening of mutations involved in various diseases [8], including MPS I [9]. We optimized an efficient dHPLC method and validated its application to mutation screening using DBS as source of biological material. This semiautomated technique results in clear profiles and facilitates the accurate detection of the p.Trp402Ter mutation in the *IDUA* gene, allowing the screening of a large number of samples. Conventional methods for large-scale detection of mutations, such as SSCP, CSGE, RFLP, and sequencing, are expensive, are technically demanding, are time consuming, and sometimes have limited resolution. The application of dHPLC allows the discrimination of mutations at a cost that is fourfold lower than dye-terminator sequencing. The presently described approach is a cost effective, sensitive, and reproducible method for screening large numbers of samples, even from distant locations, with minimal technical requirements.

## 2. Materials and Methods

### 2.1. Biological Materials

A total of 500 samples from anonymized individuals born in Portugal were randomly selected. These 500 samples consisted of surplus remainder samples of dried blood spots (Guthrie cards, DBS) from the National Program of Neonatal Screening (Instituto Nacional de Saúde Dr. Ricardo Jorge, Centro de Genética Médica Dr. Jacinto Magalhães). Appropriate approval for investigational use was obtained in accordance with national and institutional ethical regulations. Control heterozygous (GM00799) and homozygous (GM00798) human skin fibroblast cell lines with p.Trp402Ter mutation were obtained from the Coriell Institute for Medical Research. Genomic DNA was automatically extracted using a Maxwell 16 System (Promega). DNA from 3 punches (2 mm ø) yielded approximately 250 ng of DNA with a purity of 1.8 (A260/A280), and 5 ng of DNA was used as template in the PCR reaction. Differences in sample quality from gDNA extracted from different sources (fibroblast cell lines and DBS) were minimized by the dilution of the template used for obtaining the final amplicon for dHPLC analysis.

### 2.2. PCR Amplification

PCR primers were based on those proposed by Kasper and collaborators [9] with modifications, namely, removal of (GC) clamps and amplification of a smaller fragment for dHPLC analysis. Optimization of the PCR reaction was performed by varying PCR conditions including Taq mixes, cycle temperatures/times, and template concentrations.  Table 1 shows the final PCR conditions. PCR with 5 ng of template DNA resulted in clear and abundant amplicons that did not require any purification procedure. Prior to the dHPLC optimization, the *IDUA* gene of control samples was sequenced in order to ensure its integrity.

### 2.3. dHPLC Conditions

Denaturing high-performance liquid chromatography (dHPLC) analysis was performed using an automated WAVE Nucleic Acid Fragment Analysis System and respective Wavemaker 3.4 software (Transgenomic). Melt Program from Stanford Genome Technology Center (http://insertion.stanford.edu/melt) was used for the optimization of dHPLC conditions. 

Prior to dHPLC analysis, PCR products were heated to 96°C for 3 min and slowly cooled to 65°C using a ramping of 0.1°C for each 6 sec (62 cycles). Five *μ*L of the cooled PCR product was applied onto a preheated C18-reversed phase column (DNASep column, Transgenomic). DNA was eluted at a flow rate of 0.9 mL/min within a linear acetonitrile gradient consisting of Buffer A (0.1 M triethylammonium acetate (TEAA)) and Buffer B (0.1 M TEAA, 25% acetonitrile) and detected spectrophotometrically by UV absorption. 

The optimal percentage of Buffer B was 52%, run time and column temperature were 8 min and 68.5°C, respectively. After each run, regeneration of the column was achieved by washing with 100% Solution D (Transgenomic) for 0.5 min followed by an equilibration time of 2 min. To test the dHPLC capacity for detecting p.Trp402Ter, DNA from wild type and homozygote were mixed in different proportions. Samples with abnormal heteroduplex patterns, as well as randomly selected samples with normal profiles, were amplified de novo and subjected to direct automated sequencing.

## 3. Results

**Figure 1:**
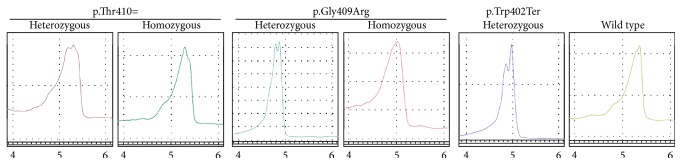
Different dHPLC profiles of the exon 9 of *IDUA* gene. Presence of heteroduplexes when the absorbance was measured against time. Differences were observed between dHPLC profiles of wild-type amplicons and amplicons carrying the p.Trp402Ter mutation or the SNPs p.Thr410= or p.Gly409Arg. As shown, retention times were 4 to 6 minutes. Profiles were normalized according to their height, elution time, and endpoints.

The optimal PCR conditions were as described in the methods section. Optimization of dHPLC conditions and subsequent dHPLC analysis of PCR products was carried out using samples with and without the p.Trp402Ter *IDUA* mutation in order to validate the technique. A total of 500 DNA samples, plus previously characterized controls, were then amplified and analyzed by the dHPLC technique. As seen in Figure 1, a variant dHPLC profile was detected in the case of a p.Trp402Ter heterozygous carrier, and four additional dHPLC variant profiles were obtained corresponding to homozygosity or heterozygosity for the SNP p.Thr410=  (NG_008103.1: g.20776C > G, NM_000203.3: c.1230C > G, and rs115790973) and homozygosity or heterozygosity for the SNP p.Gly409Arg (NG_008103.1: g.20771G > C, NM_000203.3: c.1225G > C, and rs11934801). Each of the five different dHPLC profiles was distinguishable and characterization was achieved by DNA sequencing.

The allele frequency of SNP p.Gly409Arg was 4%, whereas SNP p.Thr410= had an allele frequency of 17% (for the allelic form g.20776G), similar to the one reported in databases, demonstrating the consistent detection of alterations and the reproducibility of the assay. The dependable detection of the p.Trp402Ter mutation in heterozygous control samples showed the reliability of the assay, which was further reinforced by the finding of a heterozygous sample among the 500 randomly selected samples tested. 

The use of dried blood spots as source of DNA for dHPLC analysis was validated by the regularity of the results. 

## 4. Discussion

The existence of multiple nucleotide variations, along with high GC content and with mutations leading to decreased levels of mRNA, interferes with the efficiency of mutation detection techniques, namely, with SSCP, allele discrimination, and cDNA sequencing. With dHPLC's high level of detection, reproducibility, sensitivity, and resolution, even in GC-rich regions, we were able to discriminate between normal, mutated, and polymorphic alleles. The polymorphisms detected in this study were not detected in combination with the causal mutation p.Trp402Ter. As previously described by other groups, DBS represent an extremely convenient source of biological material [10] for mutational and biochemical screenings. This point becomes particularly important since, unlike with enzyme assays in which differences were found between DBS and leukocyte activities [11], in the case of DNA no difference was found in the results obtained from different sources of biological materials. The successful use of dried blood spots (DBS) validates the use of these samples as a reliable source of biological material for the application of the described laboratorial approach. Therefore the use of DBS is not only practical and economical but is also convenient for the large screenings or to the study of samples from distant locations. Since the molecular lesion tested is the most common causal mutation of MPS IH in Western European populations, this could be a preferable method for initial screening in populations with that particular ancestry. 

According to different reports the use of dHPLC as a mutation screening method is particularly advantageous, not only because of its simplicity and sensitivity [12] but also due to its cost, which has been estimated to be about 80% lower than that direct sequencing [13, 14]. Although the cost might vary depending on the country, in our case of it was estimated to be 3,50 euro/sample (as opposed to 14 euro per sample sequencing). Considering the use of other alternative techniques, such as SSCP or allelic discrimination, this optimized method showed a clear advantage by allowing the efficient identification of alterations in a GC-rich region while requiring very low template concentrations. 

In conclusion, with this work, we improved and developed a dHPLC screening method aiming at the detection of variations in a region of the *IDUA* gene with a GC content of 77%. This dHPLC technique was found to be a very useful screening tool since it is a sensitive, automatable, economical, and highly robust method. The combination of dHPLC with the use with DBS samples makes this approach most adequate for the screening of numerous samples.

## Figures and Tables

**Table 1:** PCR amplification conditions.

Exon 9	Amplicon size (bp)	GC content (%)	Primers (5^'^→ 3^'^)	PCR program	
First PCR	379	78	F-GGAGCGAGTGGTGGGAGG	97°C/5 min	
			R-GACACTCAGGCCTCGGCTC	**96** ° **C/1 min**	**35X**
				**60** ° **C/1 min**	
Nested PCR	208	77	F-GGCGGCTGGGCAACGACC	**72** ° **C/1 min**	
			R-GTGGGCGCGGGTGTCGTC	72°C/10 min	

PCR reactions were performed using PCR MasterMix (Promega) without any additives, 20 pmol of forward primer and 20 pmol of reverse primer; in the first PCR, 4 *μ*L (4-5 ng) of DNA was used in a 10 *μ*L final volume; in the nested PCR, 1 *μ*L of a 1/10 dilution of the first PCR product was used in a 20 *μ*L final volume.
